# Estimation of invasive Group B Streptococcus disease risk in young infants from case-control serological studies

**DOI:** 10.1186/s12874-022-01529-5

**Published:** 2022-03-27

**Authors:** Alane Izu, Gaurav Kwatra, Shabir A. Madhi, Fabio Rigat

**Affiliations:** 1 South African Medical Research Council: Vaccines and Infectious Diseases Analytical Research Unit (VIDA), University of the Witwatersrand, Faculty of Health Science, Johannesburg, South Africa; 2 Department of Science and Innovation/National Research Foundation South African Research Chair Initiative in Vaccine Preventable Diseases Unit, University of the Witwatersrand, Faculty of Health Science, Johannesburg, South Africa; 3 Statistics and Decision Sciences, Janssen Pharmaceuticals R & D, High Wycombe, United Kingdom

**Keywords:** Group B Streptococcus infections, Case-control study, Serological correlates of protection, Relative risk reduction, Absolute disease risk, Mixture modelling, Model averaging, Markov chain Monte Carlo

## Abstract

**Background:**

Group B Streptococcus (GBS) infections are a major cause of invasive disease (IGbsD) in young infants and cause miscarriage and stillbirths. Immunization of pregnant women against GBS in addition to intrapartum antibiotic prophylaxis could prevent disease. Establishing accurate serological markers of protection against IGbsD could enable use of efficient clinical trial designs for vaccine development and licensure, without needing to undertake efficacy trials in prohibitively large number of mother-infant dyads. The association of maternal naturally acquired serotype-specific anti-capsular antibodies (IgG) against serotype-specific IGbsD in their infants has been studied in case-control studies. The statistical models used so far to estimate IGbsD risk from these case-control studies assumed that the antibody concentrations measured sharing the same disease status are sampled from the same population, not allowing for differences between mothers colonised by GBS and mothers also potentially infected (e.g urinary tract infection or chorioamnionitis) by GBS during pregnancy. This distinction is relevant as infants born from infected mothers with occult medical illness may be exposed to GBS prior to the mother developing antibodies measured in maternal or infant sera.

**Methods:**

Unsupervised mixture model averaging (MMA) is proposed and applied here to accurately estimate infant IGbsD risk from case-control study data in presence or absence of antibody concentration subgroups potentially associated to maternal GBS carriage or infection. MMA estimators are compared to non-parametric disease risk estimators in simulation studies and by analysis of two published GBS case-control studies.

**Results:**

MMA provides more accurate relative risk estimates under a broad range of data simulation scenarios and more accurate absolute disease risk estimates when the proportion of IGbsD cases with high antibody levels is not ignorable. MMA estimates of the relative and absolute disease risk curves are more amenable to clinical interpretation compared to non-parametric estimates with no detectable overfitting of the data. Antibody concentration thresholds predictive of protection from infant IGbsD estimated by MMA from maternal and infant sera are consistent with non-parametric estimates.

**Conclusions:**

MMA is a flexible and robust method for design, accurate analysis and clinical interpretation of case-control studies estimating relative and absolute IGbsD risk from antibody concentrations measured at or after birth.

## Background

Group B Streptococcus (GBS) is an opportunistic commensal gram-positive bacterium colonising intestinal and vaginal flora in 10% to 40% of pregnant women [1–8]. Antenatal maternal GBS infections such as urinary tract infections and chorioamnionitis are major contributors to GBS-associated premature delivery, miscarriages and stillbirths [9]. Furthermore, maternal GBS colonization and illness are the major risk factors for invasive GBS disease (IGbsD) in newborns less than seven days age (early-onset disease; EOD) [10, 11]. Despite a historically decreasing incidence trend, GBS infections persist globally due to many factors, including intrapartum antibiotic prophylaxis (IAP) not reducing the incidence of late-onset IGbsD (occurring between 7-89 days after birth; LOD), constraints to effective use of IAP in low and middle income settings and limitations of risk-based implementation of IAP [7, 12–14]. In this context, cost-effectiveness modelling indicate that vaccination with a safe and effective vaccine against GBS in the second trimester of pregnancy in addition to IAP screening for pregnant women could prevent more GBS disease than screening-based IAP at similar cost per quality adjusted life year [15–17].

Investigation on GBS maternal vaccination in nonpregnant and pregnant women starting since the 1980s [18–29]. However, to date, no investigational GBS vaccine has been licensed, in part due to an efficacy trial likely requiring a prohibitively large number (62,000-180,000) of mother-newborn dyads to be enrolled [30, 31]. In this context, establishing robust serological markers of risk reduction against infant IGbsD is important as an alternative to traditional vaccine efficacy studies [30, 32, 33]. To this end, the association of naturally acquired serotype-specific anti-capsular antibodies against IGbsD due to the homotypic serotype has been examined in case-control studies [32]. These studies established an inverse association between the risk of EOD (and LOD) and serotype-specific anti-capsular IgG concentrations measured in maternal and infant sera; and derived IgG thresholds based on estimates of relative risk reduction (RRR) and absolute disease risk (ADR). The RRR curve estimates the relative difference between the frequency distributions of cases and controls along the IgG range. The ADR curve combines these frequency distributions with disease incidence to predict the population probability of infant IGbsD from maternal or infant IgG concentrations [34].

Published GBS case-control studies used both parametric and non-parametric RRR and ADR estimators [35–37]. These parametric models did not allow for the identification of antibody concentration sub-groups among subjects having the same disease status, and non-parametric models based on the IgG data empirical distribution admit an arbitrary number of subgroups. No study so far estimated the RRR and ADR curves allowing for clustering of the IgG concentrations of cases and of controls within sub-groups with clear clinical interpretation. Clustering is possible in GBS case-control data due to ante-natal exposure of the infant when the mother has active GBS infection manifested by urinary tract infection or chorioamnionitis, resulting in high IgG titres at birth [38]. However, accurate data on maternal GBS infection are generally hard to collect, limiting the use of analytical methods which require pre-specification of subgroups as a tool for case-control data analysis here. For example, logistic regression requires specifying the covariates which define potential subgroups and the allocation of participants into the pre-defined subgroups. Determining which covariates should be included into the model requires a model selection process and including too many covariates reduces the power to detect differences between cases and controls.

To address this gap between GBS aetiology and the quantitative models available to design and analyze GBS studies, we examine the accuracy of unsupervised mixture models using a pre-specified maximum number of components to describe clustering in the frequency distributions of IgG concentrations measured among the cases or the controls. Also, we use model averaging to account for uncertainty in the functional form of the IgG distributions and to derive robust risk estimates. We use common distributions allocating probability mass to the non-negative real line and ehxibiting exponentially-decaying right tails, including Weibull, Gamma and Log-Normal. This mixture model average (MMA) is shown here to provide flexible and accurate RRR and ADR estimates in simulated data scenarios relevant to GBS case-control studies and to clinical development of GBS maternal vaccines. Estimated MMA risk curves for two published GBS case-control studies are found consistent with non-parametric estimates and further support the hypothesis that anti-capsular IgG antibodies against GBS serotypes Ia and III protect infants against IGbsD.

## Methods

IGbsD status is denoted by the variable *D*=1 in cases and *D*=0 in controls respectively. Incidence of IGbsD in the population is denoted by *π*∈(0,1) and let *A*≥0 represent the concentration of a serotype-specific GBS anti-capsular IgG measured in either infant (including cord-blood) or maternal blood samples at or shortly after birth. The reverse cumulative distributions (RCDs) of IgG concentrations for cases and controls are denoted by *G*(*a*|*D*)=*P*(*A*>*a*|*D*) and the RRR and ADR functions are respectively:

1 $$\begin{array}{*{20}l} RRR(a) &= \frac{G(a |D=0) - G(a |D=1)}{1-G(a |D=0)},  \end{array} $$

2 $$\begin{array}{*{20}l} ADR(a) &= P(D =1 | A>a)\\ &= \frac{\pi G(a |D=1)}{\pi G(a |D=1) + (1-\pi) G(a |D=0)}.  \end{array} $$

From 1, it follows that higher RRR will be observed over antibody concentration levels associated to a wide gap between the RCDs of controls and cases, relative to the RCD of the controls. Likewise, 2 implies that greater ADR levels are the result of a narrowing of the distance between the RCDs of controls and of cases.

Non-parametric RRR and ADR curves rely on the Kaplan-Meier estimators of the IgG concentration RCDs of cases and controls. Parametric RRR and ADR have been derived in published studies from Weibull, Lognormal and Gumbel RCD models [34]. Building on these approaches, we describe the IgG concentrations in two steps to reflect more closely the sero-epidemiology of GBS. First, the IgG RCDs are defined as convex linear combinations of two components:

3 $$ {}\begin{aligned} G_{k} (a | D) &= w_{k}(D) G_{k,1} (a | D)\\&\quad+(1-w_{k}(D)) G_{k,2} (a | D)\ \text{for}\ w_{k}(D) \in (0,1), \end{aligned}  $$

with *k*=1,...,*K* indexing the 2-component model (*G*_*k*,1_(*a*|*D*),*G*_*k*,2_(*a*|*D*)) and *w*_*k*_(*D*)∈(0,1) is the proportion of subjects sampled from the *G*_*k*,1_(*a*|*D*) component. In the unsupervised setting membership of each component is unknown so that *w*_*k*_(*D*) is estimated together with the parameters of *G*_*k*,1_(*a*|*D*) and *G*_*k*,2_(*a*|*D*) from the observed case-control IgG concentrations. Since a priori it is not known what functional form will best fit the IgG data, we use the mixture model average (MMA):

4 $$ {}G(a | D)= \sum_{k=1}^{K} p_{k} G_{k}(a | D), \text{ with } p_{k} \in (0,1) \text{ and} \sum_{k=1}^{K} p_{k}=1.  $$

Here *p*_*k*_ is the weight applied to model *k* when deriving the weighted average *G*(*a*|*D*). Note that the MMA (4) can be equivalently referred to as a mixture of 2-component mixture models, with mixing distribution (*p*_1_,...,*p*_*K*_). These weights can be calculated using different methods, the central idea being that of assigning more weight to better fitting models [39–41]. Here we let *p*_*k*_ be model *k*’s marginal likelihood normalised by the sum of the marginal likelihoods of all *K* models. Each model’s marginal likelihood is approximated using the Bayesian information criterion (BIC; [42]). In this standard formulation, *p*_*k*_ represents the posterior probability of model *k* relative to the set of the *K* possible models. Note that the weights *p*_*k*_ can be alternatively used to select the best 2-component model as opposed to averaging the *K* models. Also, it is possible to generalize (4) allowing for different functional forms of the 2-component models applied to cases and controls respectively, although here we do not use this more complex model formulation. All published parametric RRR and ADR estimators are limiting cases of (4) for *K*=1,*p*_1_=1 and *w*_1_(*D*) = 1. Here we use *K*=6 models combining Weibull, Lognormal and Gamma distributions to define each 2-component mixture (see Appendix). This is a rich set of models which we find adequate to fit the case-control study data examined in this work. However, we are unable to exclude that future studies will uncover further distributional properties which will require a broader set of models. We estimate all coefficients in (4) from case-control IgG concentrations using a Bayesian framework. Markov chain Monte Carlo (MCMC) is used to calculate the marginal posterior estimates of the parameters of *G*_*k*,1_(*a*|*D*) and *G*_*k*,2_(*a*|*D*) and the mixture model weight *p*_*k*_ [43, 44]. Full details about numerical methods are provided in the Appendix.

**Fig. 1:**
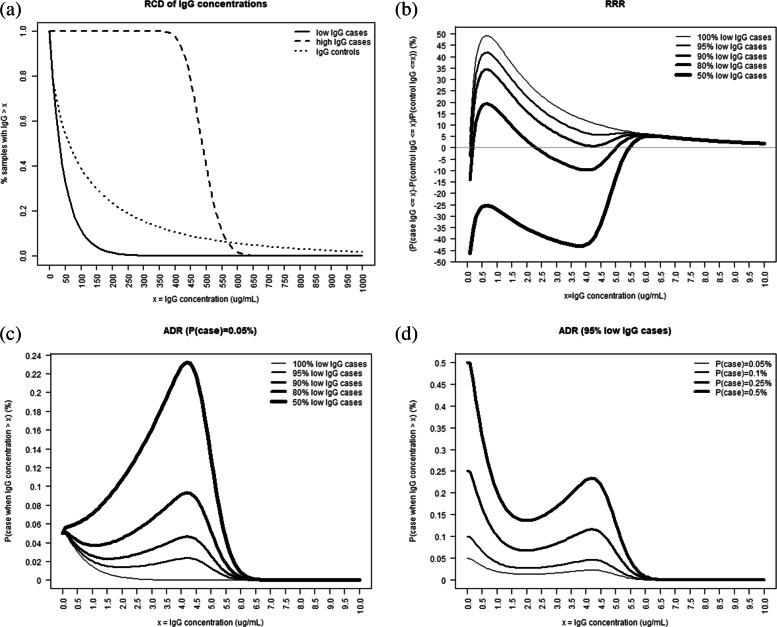
Illustrative reverse cumulative distributions (RCDs) of IgG concentrations (from infant or maternal samples) in cases and controls for antibodies associated with a reduction in IGbsD risk (panel **a**); RRR and ADR curves for different proportions of high IgG case samples (panels **b** and **c**); ADR curves for different infant IGbsD incidence values (panel **d**)

Figure 1 shows illustrative IgG concentration RCDs and disease risk curves using a simple 2-component model. The RCD of the controls (either maternal or infant samples) shown as a dotted line in panel (a) follows a Weibull distribution with median 5 *μ*g/ml.

The solid and dashed lines in panel (a) represent the IgG RCDs of two clusters of case samples modelled respectively as a Weibull distribution with median 0.5 *μ*g/ml (solid line) and a Lognormal distribution with median 1 *μ*g/ml (dashed line). The Weibull distribution is appropriate for cases with low antibody concentrations as the majority of the density mass is placed around low values. Whereas the Lognormal distribution will have most of the mass around the higher concentrations and density close to zero for low IgG values. In this model, antibodies will be associated with a reduction of risk in IGbsD when the proportion of cases from the high IgG concentration cluster is small. Specifically, when all cases are sampled from the low IgG component, panel (b) in Fig. 1 shows that the RRR decreases monotonically as antibody concentra- tions increase. As the proportion of IGbsD cases sampled from the high IgG component increases above 10%, the RRR curve becomes bimodal and shows reductions as well as increases in relative risk along the antibody concentration range. Likewise, panel (c) shows that the probability of infant IGbsD at 0.05% population incidence does not decrease monotonically in IgG concentration when high IgG cases are present. Disease risk models identifying clusters of samples will correctly show here the protective value of IgG concentrations in low IgG cases and the lack of such protection for the high IgG cases. Panel (d) in Fig. 1 shows the ADR when 5% cases arise from the high IgG component and disease incidence ranges from 0.05% up to 0.5%, covering current western world GBS epidemiology and incidence levels observed in developing countries. Since the odds of disease at the denominator in (2) decrease in *π*, the ADR increases in the overall GBS population incidence.

**Fig. 2:**
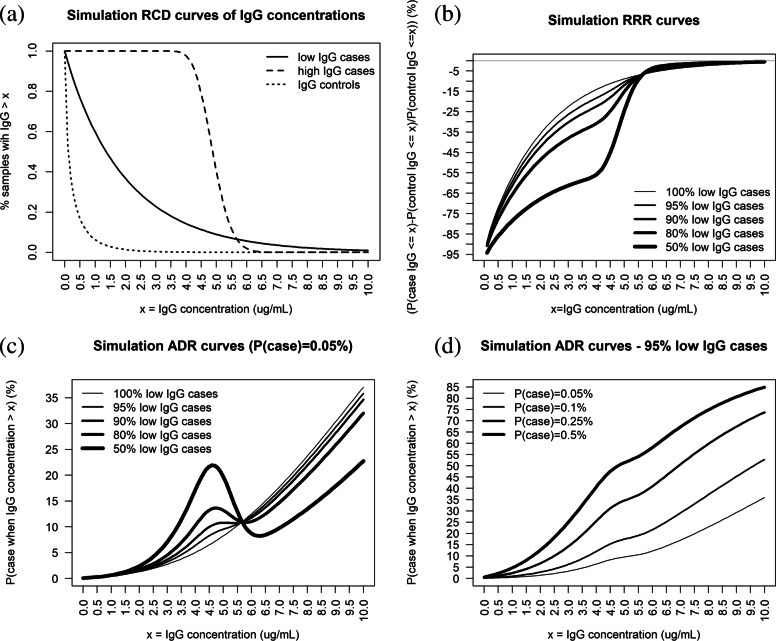
Illustrative reverse cumulative distributions (RCDs) of IgG concentrations (from infant or maternal samples) for cases and controls of antibodies not associated with a reduction in IGbsD risk (panel **a**); RRR and ADR curves of simulation data for different proportions of high IgG case samples (panels **b** and **c**); ADR curves of simulation data for different infant IGbsD incidence values (panel **d**)

Figure 2 illustrates the RRR and ADR when the antibodies have no association with a reduction of risk in IGbsD, that is when the RCD of the controls in panel (a) decreases more rapidly than those of both case clusters. The RRR in panel (b) is consistently negative and it approaches zero as all RCDs approach zero at high IgG levels. The corresponding ADR in panel (c) is increasing when the proportion of high IgG level cases is small. When this is not the case the ADR decreases over the range of the high IgG cases 4.5 *μ*g/ml-6 *μ*g/ml. Above this range, the ADR increases regardless of population disease incidence (panels (c) and (d)) demonstrating that high IgG levels in this scenario have no association with a reduction of IGbsD risk.

## Results

### In silico comparison of risk estimators

Simulation of pseudo-random IgG concentrations from the RCDs shown in Fig. 1 panel (a) was used to compare the accuracy of MMA against that of non-parametric RRR and ADR estimators. The proportions of cases sampled from the high IgG component were respectively 0%, 10%, 20%, covering scenarios favoring standard parametric models (no high IgG cases) and scenarios where a single RCD component is inappropriate (20% high IgG cases). Risk estimates were calculated from ten thousand independent simulations of 200 control IgG concentrations and the case-control ratios 1:8, 1:4, 1:2 and 1:1, reflecting the range of sample sizes in published GBS case-control studies. Accuracy of all risk estimates was measured by their mean square errors (MSEs) calculated over a grid of equally spaced antibody concentrations ranging from 0.1 *μ*g/ml up to 10 *μ*g/ml. The MSE was chosen to quantify accuracy as it measures the sum of bias and variance of the RRR and ADR estimates about their true simulation scenario values.

**Fig. 3:**
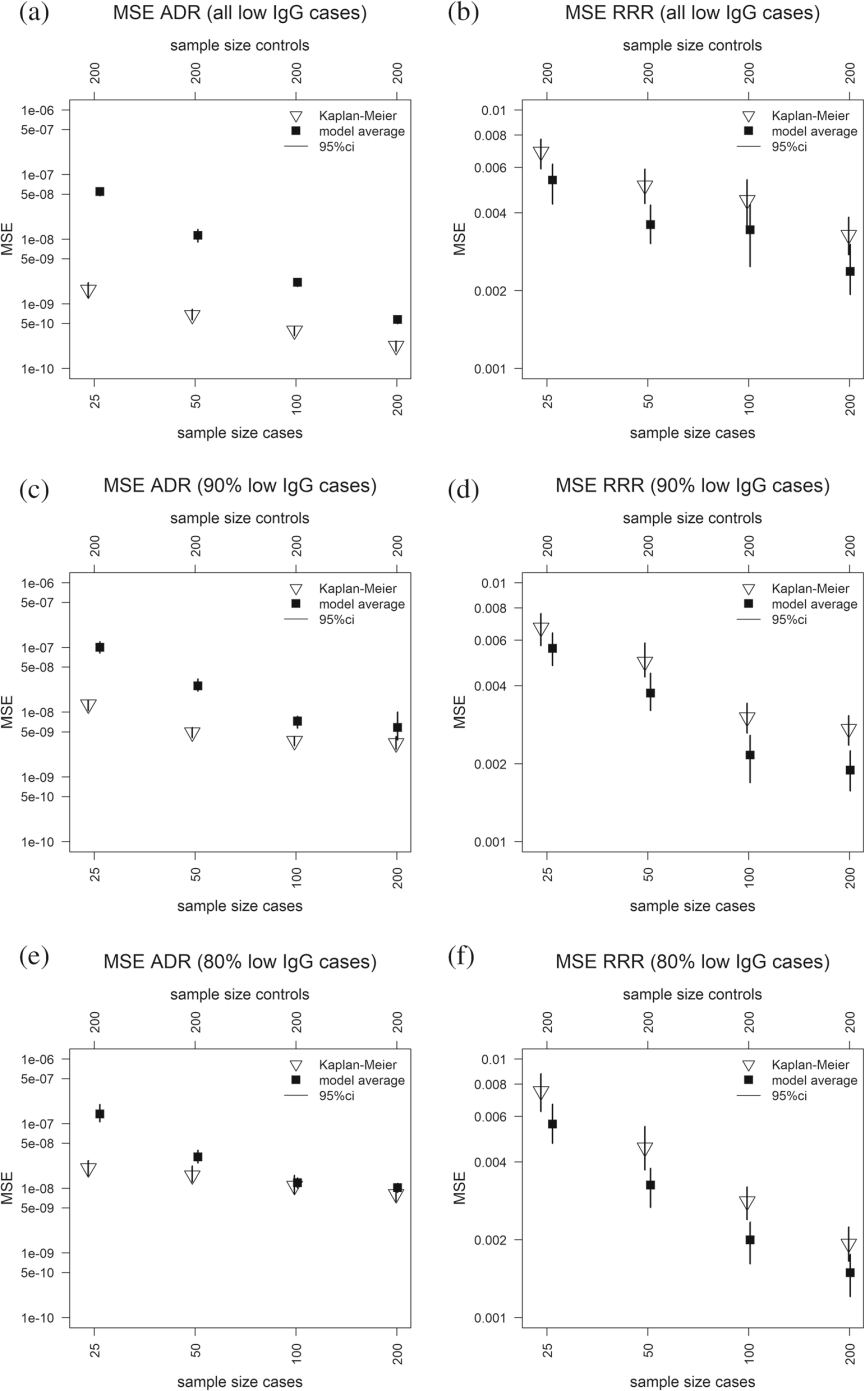
Mean square error (MSE) of ADR (left) and RRR (right) MMA and non-parametric estimates calculated from case-control data simulations

The right column in Fig. 3 shows that the MMA RRR estimates have greater accuracy than the non-parametric estimates in all scenarios. This result reflects the low bias of both MMA and non-parametric RRR estimates and a greater variability of the non-parametric RRR estimates especially at low IgG levels. The left column in Fig. 3 shows that both MMA and non-parametric ADR estimates are highly accurate in all scenarios, with the MMA estimates being at least as accurate as the corresponding non-parametric estimates when the case-control ratio is 1:2 or 1:1 or when the proportion of high IgG case samples is at least 10%.

### Re-analysis of the dEVANI case-control study

The Design of a Vaccine Against Neonatal Infections (DEVANI) consortium undertook a multi-centre sero-epidemiological study to standardize diagnosis of GBS maternal colonization and of neonatal infection, to assess disease burden and sero/genotype distribution and to inform vaccine design by investigating naturally acquired serotype-specific anti-capsular GBS antibodies in pregnant women in European countries [45]. Control samples were prospectively collected from GBS colonized pregnant women using standardized methods and harmonized protocols were used to identify IGbsD cases [46]. Maternal sera of cases were drawn either at delivery or at the time of IGbsD diagnosis in the infant. GBS anti-capsular serotyping was performed by standardized latex agglutination and anti-capsular IgG concentrations were measured by ELISA using the reference sera of Baker *et al* (2014).

**Fig. 4:**
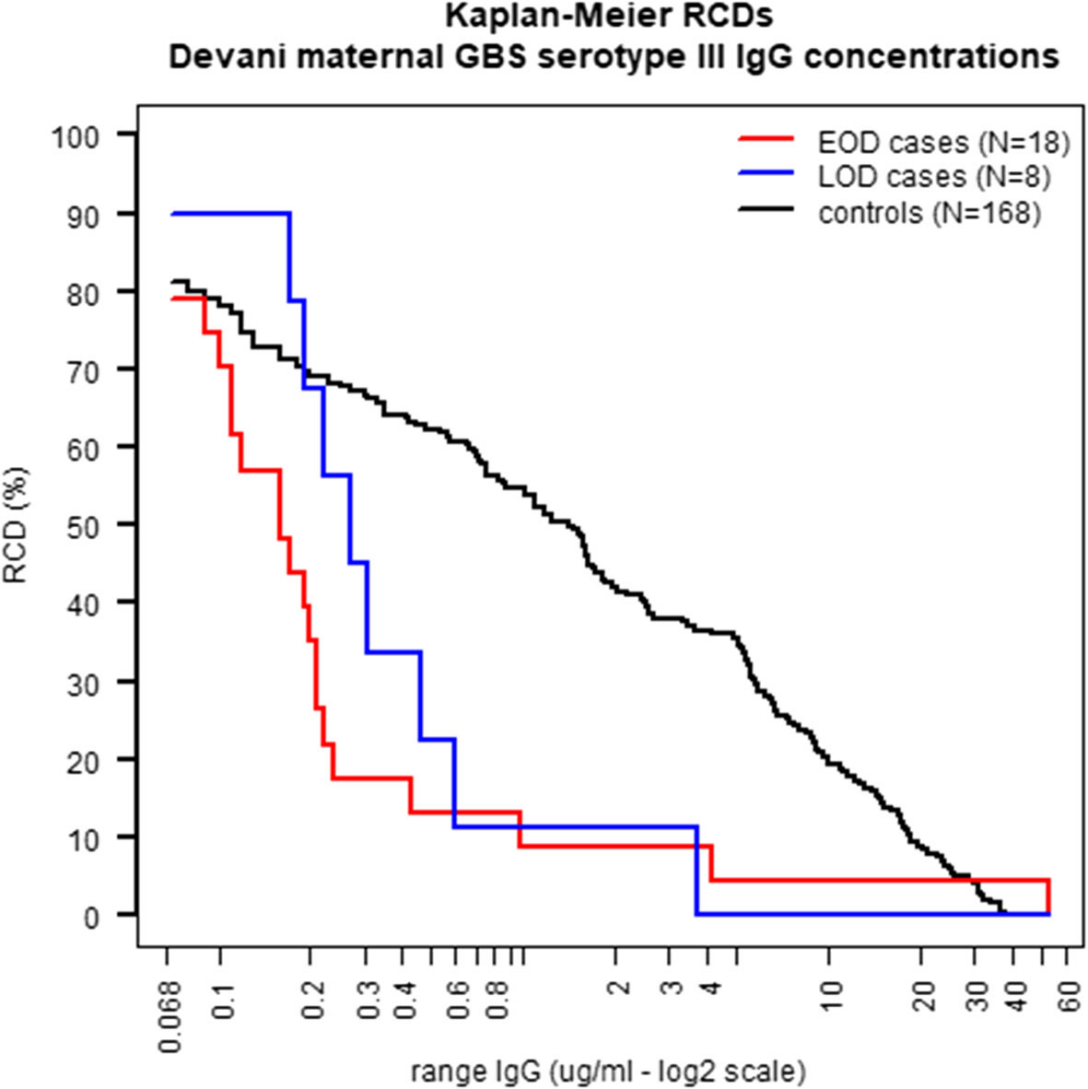
Kaplan-Meier estimates of the RCD of DEVANI GBS serotype III maternal IgG concentrations

Figure 4 shows the Kaplan-Meier RCD estimates of the IgG distribution calculated using all published DEVANI serotype III EOD (N=18 in red) and LOD (N=8 in blue) IGbsD case samples with IgG above the assay LLOQ (0.068 *μ*g/ml) [37]. The IgG concentrations of the control samples show in Fig. 4 (N=168, in black) were generated from a unit rate exponential distribution, matching the RCD of the unpublished control data. Consistently with Fig. 3-(b) in Fabbrini *et al* (2016), the RCD of the EOD cases crosses above that of the simulated controls in Fig. 4 at approximately 53 *μ*g/ml, due to the presence of one EOD case sample with very high maternal IgG concentration. It is possible that exposure of this case to GBS may have occurred in the womb prior to the development of the maternal antibodies measured at infant GBS diagnosis, but no confirmation is available. Presence of this sample in this study shows that further investigation and improved data collection are needed to determine maternal GBS infection and ante-natal GBS exposure in future studies.

**Fig. 5:**
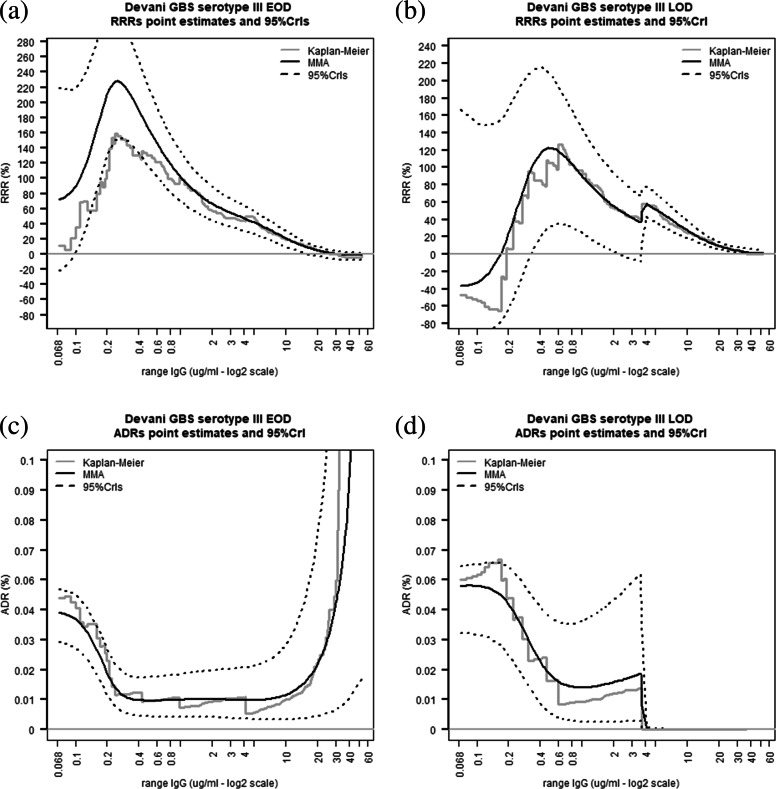
Kaplan-Meier and MMA estimates of RRR and ADR for GBS serotype III at various maternal IgG concentrations using data from the DEVANI study

The non-parametric RRR estimates in Fig. 5a and b (in grey) show global maxima at 0.25 *μ*g/ml and 0.72 *μ*g/ml for the EOD and LOD case-control data respectively, closely matching the corresponding MMA RRR maxima (0.24 *μ*g/ml for EOD and 0.8 *μ*g/ml for LOD). Figure 5a shows that a single high IgG EOD sample has little impact on the RRR estimates whereas non-parametric and MMA estimates of the ADR in panel Fig. 5c climb rapidly beyond the overall population disease incidence from approximately 40 *μ*g/ml onwards. Figure 5c and d show that infants born from GBS colonised mothers having naturally acquired anti-capsular IgG concentrations at birth against GBS III greater or equal to respectively 0.21 *μ*g/ml and 1.1 *μ*g/ml are half as likely to experience respectively early or late onset GBS infections compared to infants born from mothers having undetectable concentrations of the same antibodies. The EOD ADR increases at very high maternal IgG levels as a result of inclusion in this study of one high IgG EOD sample. This result indicates a greater sensitivity of the ADR compared to the RRR as a risk scale relevant to detecting samples needing clinical input to ascertain the potential causes of outlying antibody concentrations.

**Fig. 6:**
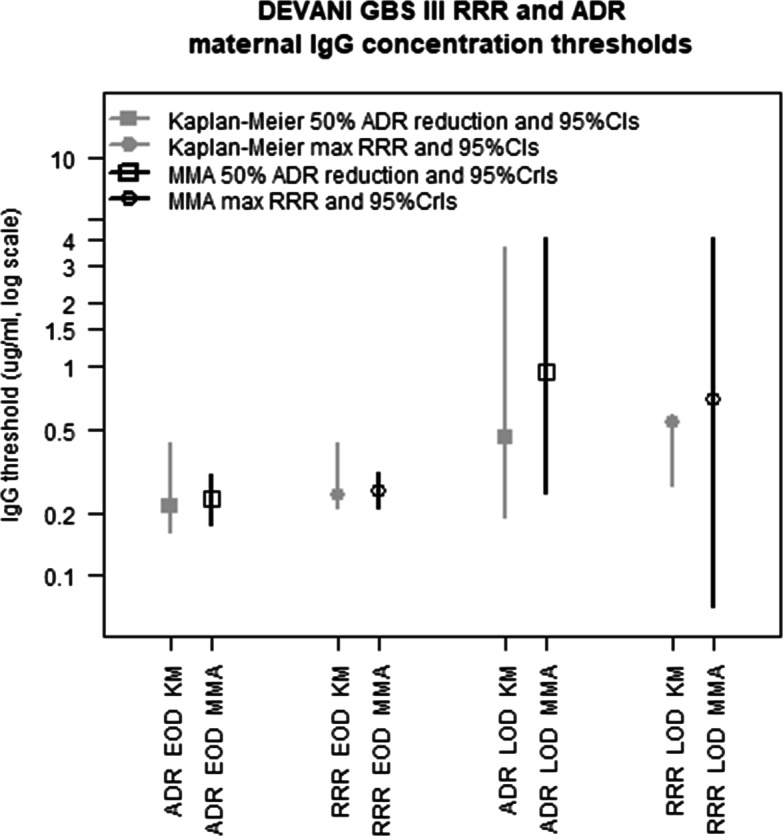
Non-parametric and MMA estimates of the DEVANI GBS III maternal IgG RRR and ADR thresholds

Figure 6 compares the non-parametric (grey) and MMA (black) estimates of the maternal EOD and LOD GBS III IgG concentration thresholds maximising RRR estimates and showing 50% reduction in the ADR. The precision of the non-parametric and MMA estimates is represented respectively by the width of their 95% bootstrap and 95% posterior probability intervals. Point and interval estimates of the RRR and ADR IgG thresholds calculated from the EOD-control data are robust to the choice of the estimation method, showing that the relatively small EOD sample size is sufficient to determine acceptably precise risk summaries. This is not the case for the risk thresholds estimated from the LOD-control data, which show notable inconsistencies between methods suggesting that the limited number of LOD samples is insufficient to identifying a robust IgG risk threshold.

**Fig. 7:**
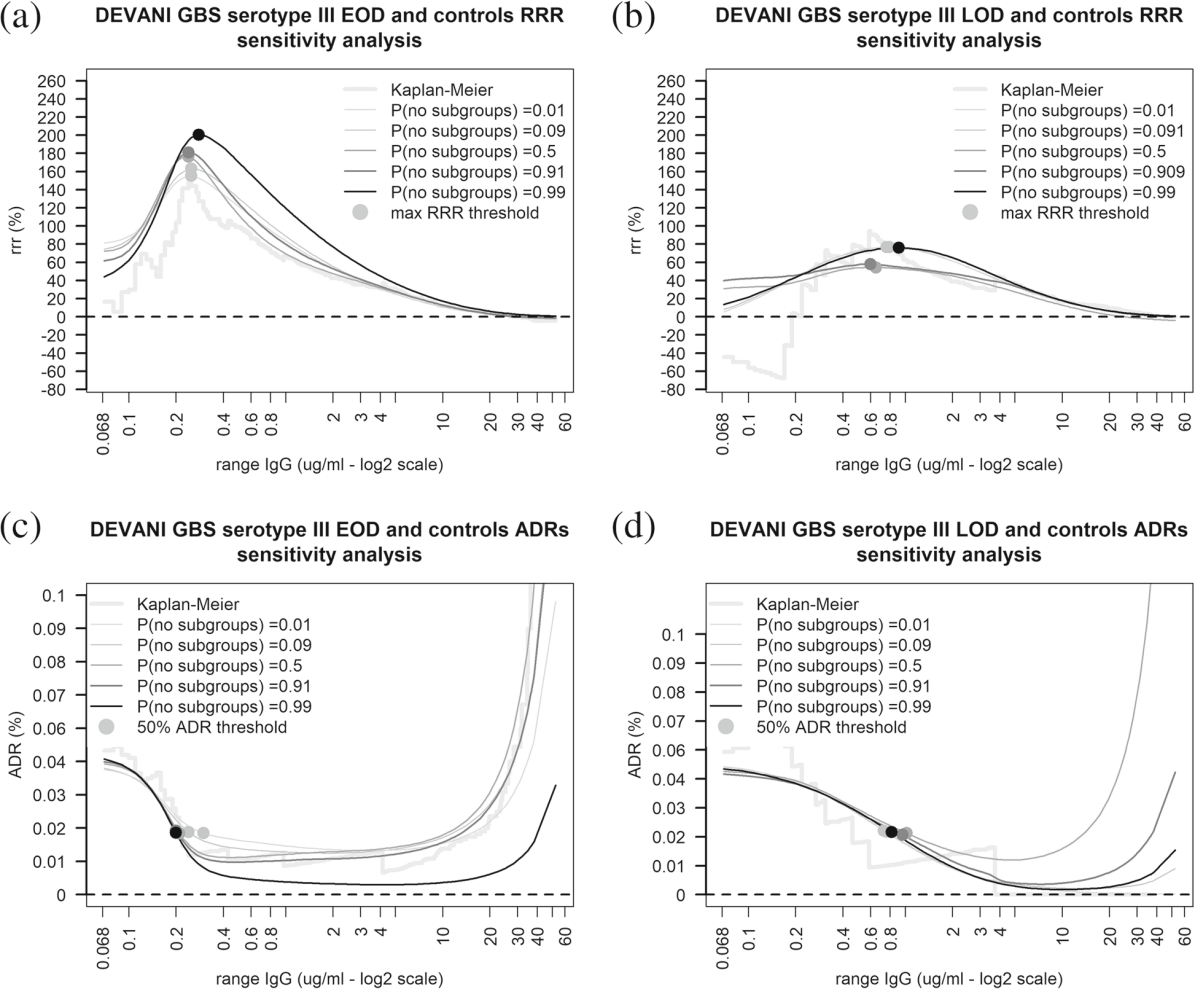
Sensitivity of MMA RRR and ADR estimates to the prior probability of IgG data clustering within a single sub-group using data from the DEVANI study

Figure 7 depicts the results of a sensitivity analysis estimating the DEVANI GBS III MMA risk estimates when the prior probability of maternal IgG data grouping within a single cluster vary over the range 1*%*−99*%*. Figure 7 shows that the IgG thresholds maximising the RRR and those associated to 50% ADR reduction vary approximately by 0.1 *μ*g/ml and 0.4 *μ*g/ml respectively on both risk scales. Figure 7c and d show that, unlike for the RRR, the ADR estimates beyond the 50% reduction threshold are heavily influenced by the prior. For the EOD data in Fig. 7c, no amount of prior shrinkage towards absence of IgG subgroups prevents the ADR estimates to increase at high concentrations consistently with the non-parametric estimate. For the LOD data shown in Fig. 7d, agreement between non-parametric and MMA ADR estimates was achieved when the prior probability that IgG concentrations cluster within a single component distribution was greater than 50%. This sensitivity analysis shows that ADR estimates of thresholds associated to 50% risk reduction is robust to prior specification, and that placing most prior probability on simpler IgG con- centration distributional forms can prevent MMA RCD estimates from showing long right tails.

### Re-analysis of the south african case-control study

Data from a South African (SA) case-control study were analyzed by Madhi *et al* (2020) to investigate the association between IGbsD and serotype-specific anti-capsular antibody measurements for serotypes Ia and III. This matched case-control study was nested within an observational study and took place at three academic hospitals in Johannesburg, South Africa between June 2016 and December 2018. The observational study enrolled all mother-infant pairs from each of the study hospitals. Maternal serum and cord blood were collected at time of birth for all participants. Vaginal swabs were taken from a subset of these mothers. Routine surveillance was used to identify IGbsD cases (positive culture of GBS from either a blood, CSF or other normally sterile site or positive CSF latex agglutination) in infants ≥0 days of age born at ≥34 weeks gestational age. Controls were infants who remained healthy up to at least three months of age that matched to cases by gestational age, maternal age and maternal vaginal colonizing serotype. A Luminex fluorescence micro-bead immunosorbent assay was used to measure serotype-specific IgG antibodies. A Bayesian parametric model was used to estimate the ADR assuming the distribution of antibody measurements for cases and controls follow a Weibull distribution. A total of 32 mother-infant pairs were enrolled as cases (Ia = 12 and III = 20) and 123 as controls (Ia= 46 and III = 77) where at least one of a maternal or infant samples was available. Further details on samples size are found in [47]. Infant IgG concentrations ≥1.04 and ≥1.53 *μ*g/ml were found to be associated with a 90% reduction in Ia and III IGbsD, respectively. Similarly, maternal IgG thresholds associated with Ia and III IGbsD reduction were 2.31 and 3.41 *μ*g/ml, respectively.

**Fig. 8:**
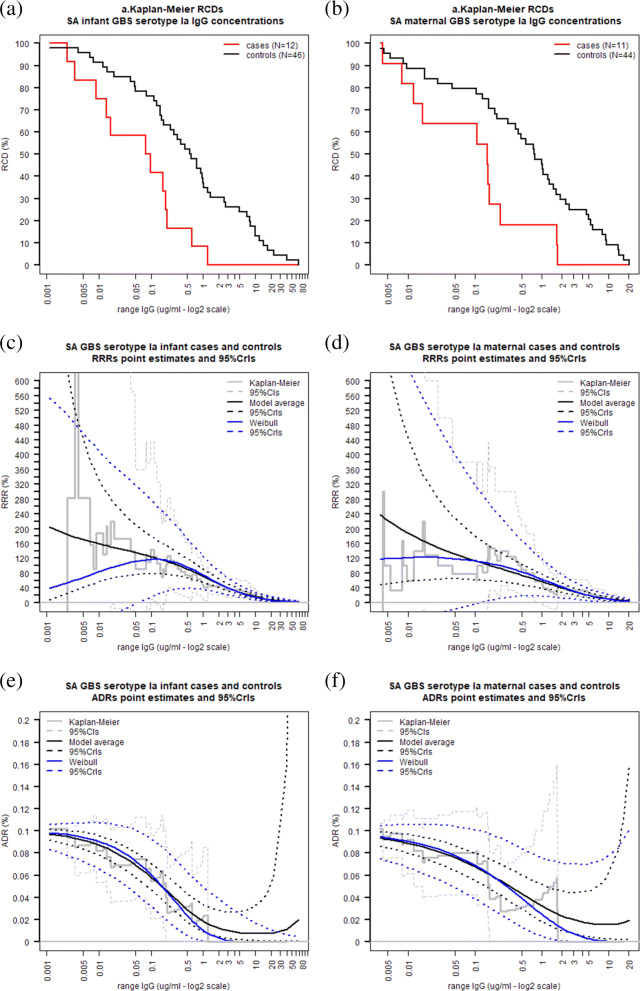
Kaplan-Meier estimates of the RCD of GBS serotype Ia IgG concentrations and non-parametric, MMA and Weibull estimates of RRR and ADR for GBS serotype Ia at various infant and maternal IgG concentrations using data from the SA study

In this section, infant and maternal antibody data from cohort cases and controls (mother-infant pairs from which samples at time of birth were available) are re-analysed and non-parametric and mixture model estimates of both the ADR and RRR are reported. The non-parametric RCDs of infant and maternal antibody concentrations for serotype Ia in Fig. 8a and b show that the RCD of the cases declines more rapidly than that of the controls. Figure 8c and d display the non-parametric, MMA and Weibull estimate of the RRR. The non-parametric estimate of the RRR is decreasing and the global maximum at 0.2 *μ*g/mL is not a clinically significant threshold. The MMA RRR is also monotonically decreasing, with estimates being similar to and more precise than those calculated using the Weibull model, as demonstrated by the smaller MMA RRR credible intervals. Figure 8e and f display the ADR estimates for the infant and maternal antibody concentrations respectively. Similarly to the RRR estimates, MMA ADR estimates here are similar to and more precise than the corresponding Weibull estimates.

**Fig. 9:**
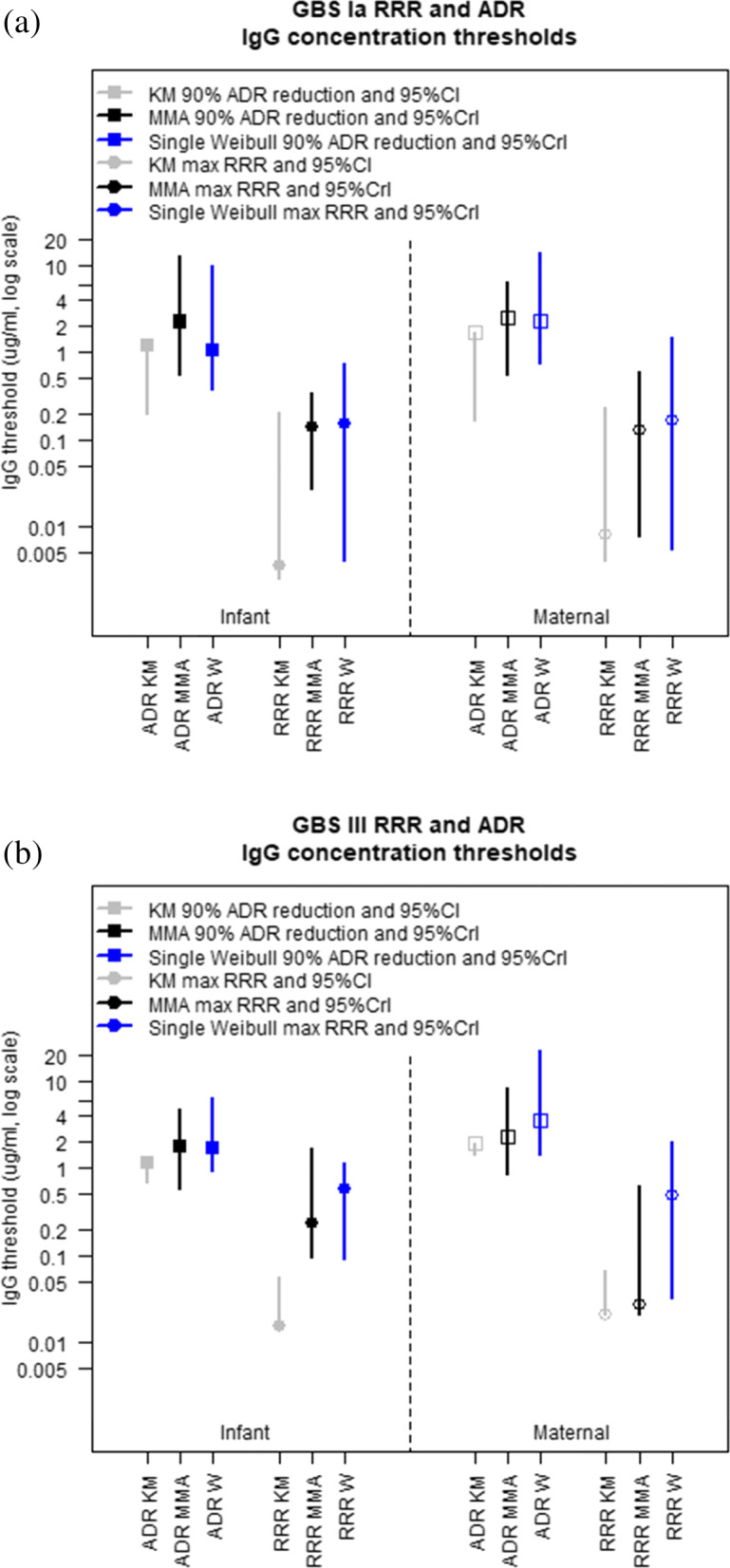
Non-parametric and MMA estimates of the IgG concentration thresholds associated with a reduced risk in GBS serotype Ia and III disease based on RRR and ADR using data from the SA study

Threshold estimates for a 90% reduction in disease risk are shown in Fig. 9. The non-parametric threshold is the maximum of the case antibody concentrations and coincides with the upper bound of the bootstrap confidence interval. When discussing the threshold from the parametric models, the number of iterations in which a threshold associated with the specified reduction in risk could not be found needs to be considered. For both mothers and infants, the minimum ADR from roughly 85% of the iterations from the single Weibull model never fell below 10%. For the MMA, at every iteration, there was at least one model where there was an antibody concentration for which the ADR was ≤ 10%. The MMA estimate for a threshold associated with 90% reduction in disease risk was the most conservative compared to other estimates, although the overlap between MMA and Weibull credible intervals of this threshold shows that their difference was not statistically significant.

**Fig. 10:**
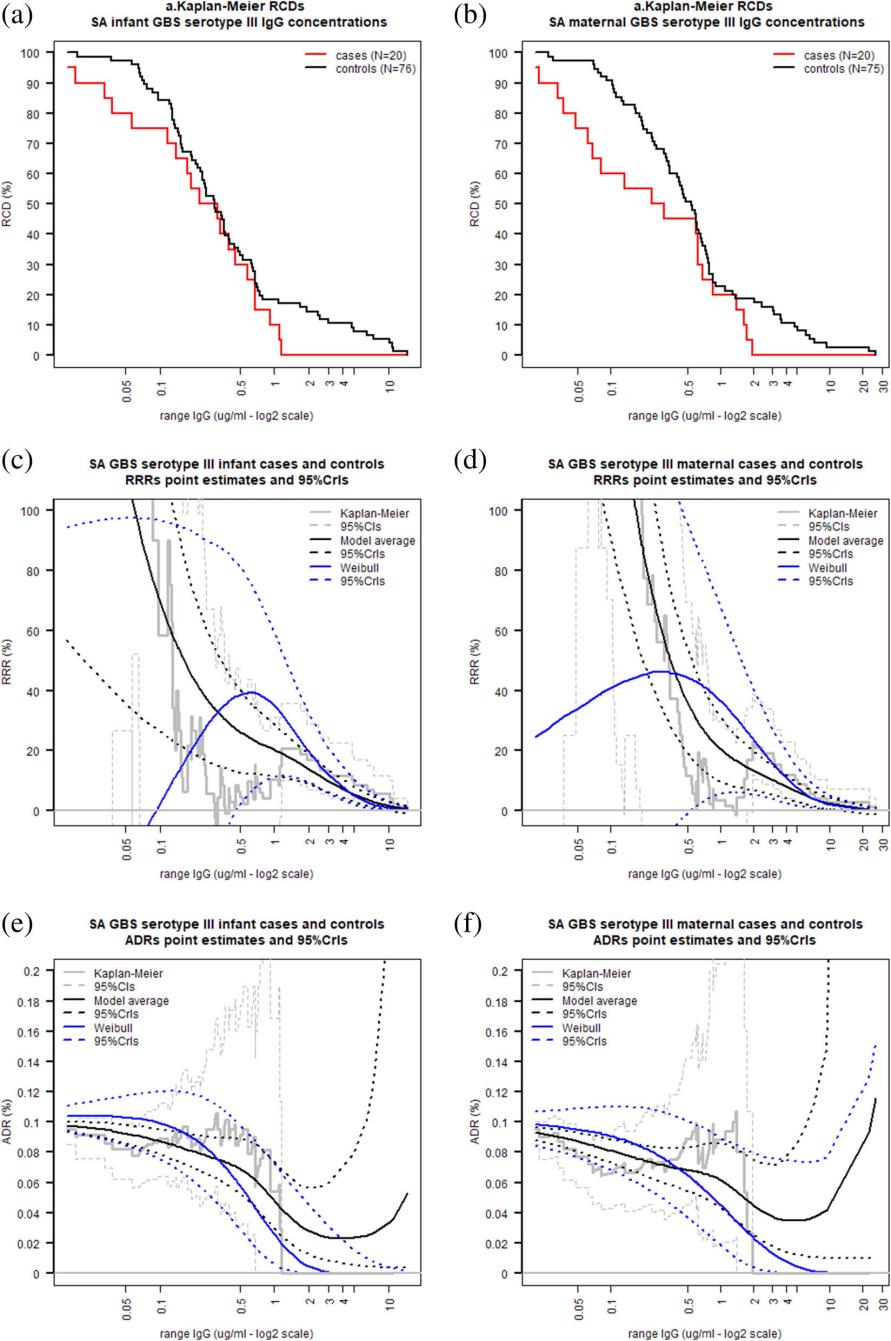
Kaplan-Meier estimates of the RCD of GBS serotype III IgG concentrations and non-parametric, MMA and Weibull estimates of RRR and ADR for GBS serotype III at various infant and maternal IgG concentrations using data from the SA study

For the South African serotype III data, the RCDs of the case and control samples shown in Fig. 10a and b are more similar to each other compared to the GBS Ia data, with approximately the same median IgG concentrations. Similar to the serotype Ia data, the MMA estimate of the RRR is monotonically decreasing and the non-parametric RRR global maximum is achieved at a low antibody concentration which is not a clinically significant threshold (Fig. 10c and d). The ADR estimates in Fig. 10e and f show that the separation between the RCDs of cases and controls is associated to a decline in the ADR. As the RCDs for both cases and controls gets closer, the non-parametric and MMA ADR estimates level off and increase rapidly at high IgG concentrations. Conversely, the ADR estimates from the Weibull model decrease monotonically and produce lower disease risk estimates at higher antibody concentrations. Here the ADR estimates from Weibull model dropped below 10% in 40% and 76% of the iterations respectively for the infant and maternal data. The associated 90% ADR reduction threshold estimates shown in Fig. 9 panel (b) range from 1.16 *μ*g/mL to 1.7 *μ*g/mL for infant concentrations and 1.95 *μ*g/mL to 3.5 *μ*g/mL for maternal concentrations, with little difference between point estimates and the MMA estimates being more precise than the Weibull estimates.

## Discussion

Accurate estimation of disease risk from case-control data is needed to inform the design of efficient vaccine clinical trials, especially when disease incidence is low. Here we observed that MMA estimates of naturally acquired antibody concentrations measured in GBS case-control studies provided estimates of infant disease risk with comparable or greater accuracy and greater clinical interpretability in relation to the aetiology of GBS infections when compared to non-parametric estimates.

Consistently with current literature, GBS disease risk was quantified on relative and absolute scales, using the RRR and ADR functions. It was noted that, were the same case-control data observed in low and high incidence settings, the RRR curve would be identical and the ADR curve would be scaled to the overall disease incidence. Estimation of the RRR is thus unable to directly inform policy making because it does not quantify the potential impact on disease incidence of interventions changing anti-GBS antibody levels in pregnant women. It was also noted that the ADR curve is more sensitive than the RRR to the rate of decay of the antibody concentration distributions at high IgG concentrations. Qualitative consistency between non-parametric and MMA estimates of the ADR was then used as a basis for MMA prior calibration, to control the ADR estimates at high IgG concentrations where data is scarce and inference will be less robust.

Reanalysis of DEVANI GBS III case-control study data showed consistency between MMA and non-parametric disease risk estimates. Maternal antibody concentration thresholds of approximately 0.2 *μ*g/ml and 1 *μ*g/ml were found to be associated with a risk reduction in EOD and LOD IGbsD, respectively. It was alse observed that the main bottleneck to furthering the understanding of infant IGbsD risk in observational studies is a lack of specific and accurate data on occult GBS maternal infection (e.g. urinary tract infection or occult chorioamnionitis) in the case-control setting. Further work is needed to improve study design and data collection, including a more detailed characterization of chorioamnionitis, of intrauterine infection and of GBS-specific manifestations of maternal infection.

The reanalysis of data from a South African study presented an example using relatively small sample sizes and similar IgG distributions between cases and controls. Both non-parametric and parametric RRR estimates were poorly informative in deriving a threshold associated with a reduction in disease. The MMA provided the most precise estimates for the ADR. At high antibody concentrations, the non-parametric, MMA and Weibull estimates were substantially different and their precision was low, yielding differing estimates of their associated 90% GBS infant disease risk reduction thresholds. This variability shows a need for careful planning of case-control sam- ple size when inference is needed for disease reductions greater than 50%. If these protective thresholds will be further confirmed, they will provide added confidence in the possibility that disease caused by GBS may be prevented by passive protection of infants through maternal vaccination. Should these interventions be safe and effective, their impact on later maturation of the infant immune system and on response to early routine vaccination will need to be thoroughly characterized [48].

## Data Availability

Devani EOD and LOD data are available in the Supplementary Materials of [37]. SA data will be shared upon request from AI on a collaborative basis. Computer code implementing simulation and MMA estimation of the RRR and ADR curves will be made available by the Authors as a freely distributed R code package (https://cran.r-project.org/).

## Abbreviations

GBS: Group B streptococcus
EOD: Early onset disease
LOD: Late onset disease
RCD: Reverse comulative distribution
RRR: Relative reisk reduction
ADR: Absolute disease risk
MCMC: Markov chain Monte Carlo
